# The job performance and job burnout relationship: a panel data comparison of four groups of academics’ job performance

**DOI:** 10.3389/fpubh.2024.1460724

**Published:** 2025-01-03

**Authors:** Miao Lei, Gazi Mahabubul Alam, Karima Bashir

**Affiliations:** ^1^Student Affairs Division, Yancheng Teachers University, Yancheng, Jiangsu, China; ^2^Department of Foundation of Education, Faculty of Educational Studies, University Putra Malaysia, Serdang, Selangor, Malaysia; ^3^Department of Education, Faculty of Education, Kebbi State University of Science and Technology, Aliero, Kebbi, Nigeria

**Keywords:** China, job burnout, competence, measures, university academics, psychological counseling, job performance

## Abstract

**Objectives:**

This present study investigates whether performance can influence job burnout, and it further examines whether there is a meaningful difference in the association between job burnout and job performance in universities. Provided here are applicable strategies aimed at preventing and maximizing job burnout crises before the job is taken and during its execution.

**Methodology:**

To answer the research questions quantitatively, group regression analysis utilizing panel data from 2020 to 2023 was employed. The instruments include the KPI and mental health records to evaluate the level of job performance and job burnout. Likewise, a total of nine universities were purposively and randomly selected, and 1,113 academics were sampled for the study. The KPI scores and frequency of burnout counseling was collected from the human resource department and the medical health centers.

**Findings:**

The results showed that academics’ job burnout is influenced by their job performance (*β* = −0.014, *p* < 0.001). Academics’ superior performance was notably linked to lower job burnout and the need for psychological counseling. Furthermore, academics’ job burnout was significantly moderated by psychological counseling (*β* = −0.006, *p* < 0.05), and neither did it regulate their job performance.

**Conclusion:**

Academics with high performance levels exhibit low levels of burnout. Meanwhile, academics who demonstrate low or poor performance indicate high burnout levels. Psychological counseling can moderate the level of job burnout but does not cure burnout. This study suggests that competency is the basic bedrock for strong performance and less burnout experienced by staff. Consequently, all universities should employ their staff based on assessing their competency and ability to handle stressful situations to prevent job burnout crises from occurring.

**Implications:**

This paper makes a contribution to the existing literature on job performance and job burnout by utilizing a distinctive measurement path approach. In this context, universities need to use pre-measurement mechanisms to prevent burnout instead of post-measurement techniques through proactive recruitment strategies based on the popular adage that “prevention is better than cure.”

## 1 Introduction

“Job burnout” as a concept is understood to be a multifaceted construct with multiple characteristics that are mostly connected to stress and work. Burnout is defined as a chronic condition and closely linked to a stress-related syndrome which results in emotional exhaustion, cynicism and depersonalization (1, 2). While studies on job burnout had their origins in social and historical perspectives, the subject has become more psychological over the centuries with greater emphasis being paid to medical experience and practices (1, 3). However, the research findings which revealed strategies and approaches to addressing job burnout to ensure staff members’ wellbeing, ignited a surge of theoretical exploration in the areas of organizational administration and management and staff welfare (4).

### 1.1 Research motivation, significance and novelty

The higher education landscape has hugely changed due to the last few decades of the sector’s internationalization and nations’ investment in it, which has led to two important outcomes: firstly, increased demands on staff; and secondly, intensified pressures to deliver high-quality services (5). There is subsequently an increasing number of studies regarding the effective management of higher education and the courses or programs that are taught within it. For example, Parmar et al., Amer et al., and Watts and Robertson (6–8) have outlined what they believe to be the primary causes and reasons (such as, turnover intention, work overload, and inefficient management) for job burnout among academics. Asfahani and Lei et al. (9, 10) have observed the correlations between job burnout and higher education in role conflict, job autonomy and emotional job demands, and concluded that job characteristics wield a significant impact on burnout.

Several studies analyzed other predictors of burnout such as social support (11), employee turnover (12) and staff engagement (13), etc. Leiter, Maslach, and Liu et al. (14, 15) added the element of incompetence, which is thought to gravely impair employees’ job performance. It is in fact the main cause of their job burnout, even though, they agreed with previous studies identifying the reasons for why job burnout occurred.

In order to address this persistent challenge, Maslach et al. and Nahas (1, 16) put forward some solutions to job burnout which include the following: adequate resource allocation, motivation and counseling, resilience, and balancing family, social, private and work life among others. Yet, the job burnout crisis remains unresolved with new cases emerging and increasing throughout the world. According to Blaskova et al. and Fazey and Fazey (17, 18), teachers, administrative staff and students in the university sector lack the essential skills, knowledge, and motivation needed to develop and effectively navigate their environment. If this is actually the case, the current recommended strategies for addressing job burnout may not be very effective, since higher education institutions are better off thinking more about pre-measurement strategies before burnout occurs, rather than considering post-measurement strategies after the job burnout crisis occurs. Subsequently, this study is motivated by exploring the ongoing challenges, aiming to delve into the scope of the problems and offer alternative strategies.

Studies have identified that job performance is predicted by job burnout, and the level of job burnout greatly influences the level of job performance (19). Unfazed by these findings, this study re-directed the path for the analysis and argued that staff members’ performance should determine the level of burnout—a novelty of this research and a methodological contribution to future studies on job burnout. Whether there is a meaningful difference in the relationship between “job performance level” and “job burnout” is still a subject of much debate. Answers to these specific aims can significantly enhance the literature on job burnout by providing more details and perspectives on the issue. Similarly, this research contributes to the literature on this topic by providing solutions that can help to prevent job burnout among academic staff, thereby enhancing staff wellbeing and universities’ effectiveness and efficiency.

This study has not only contributed by increasing the knowledge on job performance, but provided a mechanism for academic staff recruitment that can ensure sustainable high performance by all staff. Furthermore, the managerial implications of this research are not limited to universities, as staff of other institutions including non-educational sectors (such as health and social work) can also prevent job burnout by using these strategies. Moreover, the current research is empirical and quantitative in nature, comparing among various types of performance groups, and uses secondary data that are more reliable than perceptions of respondents (20). Thus, the questions that follow are intended to clarify these objectives:

1. Can the academic staff members’ job performance influence their job burnout?
2. What strategies can be created to tackle job burnout crises experienced by academics?

The remaining sections are organized as follows: first, we provide the literature review, followed by an explanation of the research design and the data used in this study. After that, we will discuss our results before making substantial conclusions.

## 2 Literature review

### 2.1 The JD-R model

The JD-R model has been extensively implemented to interpret job burnout in a range of scholarly fields (3). Bakker and Demerouti (3) created the JD-R model in an attempt to understand the factors that lead to the problem of burnout. The model divides all job traits or characteristics into two groups: firstly, job demands and with job resources; and secondly, job resources which to some extent buffer the role of job demands in triggering job burnout. In this context, valuable job resources that individuals strive to acquire or maintain (increased job opportunities, colleague support) can reduce burnout, or buffer the link between job burnout and job demands. Current research strongly suggests that job performance can be viewed as a resource that individuals strive to obtain in order to produce expected results or minimize negative consequences such as burnout. Employees who care about the progress they are making in their career or ability to improve their day-to-day tasks, will seek ways to manage job pressure and resolve negative outcomes, striving to perform as best they can in an effort to ensure their potential promotion or recognition for the work they do.

This study proposes that employees’ job performance is a precursor rather than a result of job burnout, which helps to extend the research on job burnout. Due to previous research mainly focusing on identifying sources of job pressure and recommending the usage of organizational resources to mitigate employees’ experience of job burnout (3), this study establishes guidance for academics to seek personal answers (rather than organizational answers) to address burnout. The following section introduces the relevant literature and formal hypotheses.

### 2.2 Job burnout in higher education

For nearly 50 years, job burnout has been extensively studied as a multi-dimensional construct closely linked to on-going work-related stress (1). Maslach and Leiter (2) defined it from a psychosocial perspective; they claimed that it is a chronic stress syndrome characterized by depersonalization or cynicism, emotional exhaustion, and a much reduced sense of personal accomplishment/achievement or efficacy.

In recent decades, academics in higher education worldwide have faced growing demands, including increased pressure to publish in journals, achieve high rankings, secure tenure, and maintain excellence in teaching and supervision. These pressures have increased the cases of academics’ burnout. The number of academics has expanded significantly as a consequence of the development of mass higher education systems and frameworks in emerging nations; figures show that between 1980 and 2018, the number of academics increased by about 9 million (21). Watts and Robertson (8) contended, however, that an increase in quantity often differs from the increase in quality. Furthermore Amer et al. (7) opined that there are persistent job burnout crises in universities due to a lack of competency.

China offers a noteworthy example of how its higher education system has changed over the last few decades. Since 1999, the nation has experienced a swift transformation of its university sector, evolving from an elite model to one of mass participation (22). From 2000 to 2021, the number of universities expanded from 1,000 to 2,756, and the academic staff grew from 0.46 million to 1.87 million. This shift resulted in China becoming of the world’s countries with the world’s largest education population. In this context, it is essential to examine what has caused the wave of burnouts in China’s vast higher education sector. It is also important to identify precautionary measures that could mitigate burnout, ultimately enhancing university management.

### 2.3 Job performance in higher education

In higher education, “job performance” encompasses all employee responsibilities and behaviors that carry an expected value, whether positive or negative. This speaks to employees’ performance of their jobs and conduct at work (23). Therefore, the performance of all employees as a whole determines how well the university functions (24) and universities evaluate employee performance and reward roles and good behaviors associated with excellent instruction as well as organizational regulations (25, 26).

For assessing job performance, the Key Performance Indicator (KPI) model is now the most widely recognized approach (27). According to this approach, staff duties are divided into micro-units, each of which has goals and a score. Numerous stakeholders are involved in the scoring process, which assigns points based on both quantitative and qualitative factors in a hierarchical sequence (27). Despite criticism as a “numbers game,” the KPI model is employed throughout the world by higher education institutions (28), including China.

In China, the KPI result is a combination of self-reports (i.e., subjective measures) and supervisor/colleague ratings (i.e., objective reports), wherein the reported association between different sources of performance assessment refers to two mentioned stakeholders to reflect fairness (29). Each academic has to submit his/her self-evaluation based on their KPI completion status, such as teaching, research, publications, social services, virtues, and management roles. Each item has a detailed score, with the highest score considered excellent and the lowest score deemed to be unqualified. Based on the meetings’ outcome, the supervisor/head of the academics or chairperson will make a final assessment and evaluation. This task is usually summarized by the personnel or human resources departments of each university at year’s end.

Prior to the KPI, how well the employee performed was evaluated using the Individual Work Performance Questionnaire (IWPQ) (30) and the Annual Compensation Review (ACR) (31). The IWPQ was developed to address the limitations of ACR, but it also could not capture the realities of the staff performance, and it failed to give account for the differences in hierarchical levels. The fundamental theoretical performance test may not accurately reflect reality or what their duty statements required, which is another reason the IWPQ failed (32). Meanwhile, ACR was condemned due to its biases (nepotism, prejudice, favoritism) and being influenced by managers who play a crucial role in evaluating staff members’ performance using descriptive analysis that was based on job descriptions (30).

A search was undertaken in the Web of Science core collection using the term “key performance indicators,” and it resulted in a total of 20,780 articles. Cozzani Valerio and Anil Kumar, as experts in this field, have H-indices of 49 and 43, respectively. In the study, papers emphasize the importance of KPI in the development process of enterprises, due to its flexibility and efficiency, and gradually transitions to use in universities. In recent years, more than 3,000 papers have been published on the application of KPIs in higher education. Furthermore, there are 242 articles on ACR and 5,248 articles on IWPQ, respectively. Consequently, for the purposes of this study the KPI model score which is currently the mainly official performance measurement method used in China for appraising academics’ job performance will be utilized.

### 2.4 Job burnout and job performance

The term burnout has now enjoyed methodological and conceptual analysis recognition and legitimacy for more than half a century. The approach that dominated the 1970s was mainly qualitative, employing narrative and phenomenological approaches aimed at predicting the cases of “job burnout” (3, 33, 34). Later, studies conducted in the 1980s employed a quantitative approach, testing probable hypotheses and analysis models to predict job burnout (1). Currently, comparative study of burnout and non-burnout groups and correlational/causal links between burnout and other factors strongly imply there is a negative relationship between job performance and job burnout. The scholarly literature on job burnout has been dominated by this relationship in recent decades. Similarly, some research is conducted using mixed methods (combining qualitative and quantitative approaches). One critique/limitation of this strategy is that it has always typically been used to study job burnout and their job performance in the workplace, and a call for new approaches has been made (1, 35).

**Figure 1:**
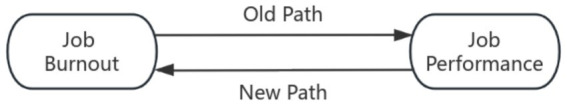
Indicating old path and proposed path.

Leiter, Maslach, and Lei et al. (14, 36) also raised concerns that job burnout could be predicted or affected by individuals’ performance. Poor performance as an after-effect of job burnout may have been erroneously documented by the relevant literature (1, 19). To achieve this, “job burnout” served as the independent variable while “job performance” became the dependent one. The focus of this study is to create a new paradigm by examining the link between the dependent variable, i.e., job burnout and the independent variable, i.e., job performance (see Figure 1).

As a result, job burnout is a consequence of their performance. To determine whether job performance affects job burnout, a comparison will be made among different job performance groups, while controlling for factors contributing to burnout.

Ha1: When all predictor factors are included, the “job performance” of academics has a significantly negative effect on “job burnout.”

The World Health Organization’s (WHO) has defined burnout as a workplace illness that can lead to potentially fatal medical disorders including depression and cardiovascular disease (37). Similarly, medical treatments and psychological therapy were suggested by the World Health Organization (38) as the approved methods for treating job burnout in the workplace. However, techniques adhering to the “prevention is better than cure” motto (4, 39), were not duly respected. Hence, this research hypothesizes that:

Ha2: When all predictor factors are included, academics’ “job performance” and their “job burnout” are significantly moderated by “psychological counseling.”

The group’s academic performance was assessed to support the claim that having employees who are more competent reflects their ability to be resilient to job burnout when all predicted factors connected with job burnout are taken into account. It is therefore important to ensure that highly competent staff are recruited as a preventative measure against potential job burnout issues and to use psychological counseling as a treatment for burnout. This study will investigate whether implementing these preventive and supportive measures significantly reduces job burnout.

## 3 Materials and methods

### 3.1 Research design

This quantitative study is a correlational design, which has undertaken a diversification and rerouting of data and their analysis compared to the recent research conducted by Lei et al. (36, 40). In doing so, the study has accumulated a panel dataset to explore the association between job performance and academic staff members’ burnout in universities. Using a panel dataset for analysis offers both time-series and cross-sectional data, which serves to enhance the richness and diversity of the dataset. According to Maslach et al. (1), there are numerous antecedents influencing job burnout, making it impossible for this study to list all control variables. Panel data with the fixed effect model (41) makes it possible to observe changes that occur within the same group of staff at different periods and control for individualistic heterogeneity, which means controlling for unobserved time and distinct characteristics that may affect the results. This would provide more accurate analysis results. Based on the model, it is suitable for variables at different analysis levels (i.e., universities, regions) in multi-level or hierarchical modeling, which is relevant to the sampling introduced below. According to Hsiao (42), this method is better suited for detecting dynamic hierarchical relations between variables, uncovering causal links and long-standing effects.

In addition, to identify the best model, *F*-test was employed to find out if the fixed effect model is superior to the pooled regression model. According to Table 1, the *F*-test result shows *p* = 0.000 < 0.05, meaning that the fixed effect model is preferred over the pooled regression model. To ensure the accuracy of the fixed effect model and eliminate estimation bias caused by multicollinearity among variables, a multicollinearity test was conducted. If any VIF value exceeds 5 or the Tolerance value is less than 0.1, a multicollinearity issue is indicated. According to Table 2, all VIF values are less than 2, and all tolerance values are greater than 0.1. This strongly suggests that no multicollinearity problem exists among the variables and that the model estimation results are valid.

**Table 1:** *F*-test.

Type	Objective of the test	Test value	Test conclusion
*F*-test	The comparison between the fixed effect model and the pooled regression model.	*F* (1,112, 2,225) = 1.244, *p* = 0.000	The fixed effect model

**Table 2:** Collinearity test.

Variables	VIF	Tolerance
Job performance	1.001	0.999
Gender	1.000	1.000
Age	1.000	1.000
Years of employment	1.001	0.999

VIF, Variance Inflation Factor.

### 3.2 Measures

The data obtained from the selected universities include both demographic information, KPI results, and burnout records as well as the receiving of psychological counseling, all belonging to the same respondents. “Processed” secondary data also known “treated data” was collected from selected institutions with the assistance of the head of the human resources department or their assistant, which is considered more reliable than survey responses for the first study in its kind in China. Therefore, since the data exists as processed secondary data, the sampled universities are responsible for the data and therefore no reliability and validity testing has been conducted.

#### 3.2.1 Control variables

Individual characteristics of the participants (age and gender) and work-related factors (years of employment) with established associations with job burnout (43, 44), are identified and controlled.

#### 3.2.2 Job performance (KPI) tool

The job performance of the academics was determine using KPI. The KPI helped to evaluate how well the academics did their work. It is compulsory for universities in China to utilize specific KPIs that will guide the ranking of staff members’ job performance. Some specific details of the KPI measurement items include teaching, research, publications, social service, virtue and managerial roles. This process is based on the national policy (45). Another policy (46), in general, according to the quantity, quality, timeliness, and cost of completed work, as well as the resulting social and economic benefits, the academic achievement is divided into four categories: non-performance which also refers to unqualified, low performance also known as basically qualified, average performance which is termed as qualified, and finally high performance which is conceptualized and graded as excellent. If academics achieve the performance tasks for the current year, they are considered to be qualified. Exceeding these performance tasks will result in an evaluation of excellence. Conversely, failure to meet the performance standard will lead to a rating of either basically qualified or unqualified.

#### 3.2.3 Psychological counseling tool

Each institution’s Mental Health Centre supplied additional data on psychological counseling. Concurring to health policy (47), all universities and higher education institutions are mandated to often provide academic staff with psychological health care, psychological assessments, and related/extended services. Universities will offer psychological treatment at the proper frequency based on the burnout results. Table 3 provides specifics on the measures.

**Table 3:** Tools and their measurements.

Variables	Tools/instruments	Domains
Independent variable Job performance	Key Performance Indicator (KPI)	Non-performance-1 Low performance-2 Average performance-3 High performance-4
Moderating variable Psychological counseling	Mental Health Centre records	Non-psychological counseling-1 Monthly Sessions-2 Bi-Weekly Sessions-3 Weekly Sessions-4
Dependent variable Burnout	Mental Health Centre records	Non-burnout-1 Low burnout-2 Moderate burnout-3 High burnout-4

#### 3.2.4 Burnout level tool

The data for measuring burnout level was adopted. This data was obtained first-hand from the Mental Health Center of each selected institution sampled for this research. The health centers used various burnout instruments and tools such as the burnout inventory scale by Maslach (1), Oldenburg (48), Copenhagen (49) and many more. This depends on the preference, rationale and justification of the health center personnel/professionals to determine whether an academic is burned out or not. Similarly, they used their clinical processes to categorize academics based on their burnout level. The research used a scale to categorize burnout levels into four distinct classes so that the extent of academics’ burnout could be documented. Thus, no reliability and validity test were performed since the data is secondary data.

### 3.3 Data collection method

This study was conducted between January 3 and June 13, 2024. All participating academics were required to be actively working between 2020 and 2023 to highlight their multi-year career trajectories. Exclusion criteria applied to those who failed to participate in the KPIs or missed a KPIs during the 2020–2023 period. Therefore, the academic staff who had were absent or retired were excluded from the study. This strict selection criterion helps ascertain the research findings’ reliability and validity.

The secondary data was collected through sampled universities. The first author approached the sampled universities and explained the study’s goal to the appropriate authorities and the specific data being requested. Prior to conducting the official visit, the research team got an ethical approval from one university with reference number: JKEUPM-2023-676, wherein the concerns regarding the safety aspects of the research had been addressed. Afterwards, authorization was acquired from every university and the institutions approved and the researchers were furnished with a reference number (YCTU20221017). The universities chosen were subsequently informed that participating for them was optional, and furthermore they have the freedom to withdraw from the study at any point.

In order to ensure that all participants’ details remained confidential, we allocated number codes in a sequential manner, commencing with the initial sample from the list. Since the data obtained from the sampled universities identified the respondents as 1, 2, 3, 4, 5, 6 and so on, all the volunteers’ personal information remained anonymous and not disclosed to the authors. Moreover, letters of the alphabet starting from A were allocated to the universities in a sequential order, based on the list of the universities. For example, the code assigned to respondents from university A, B, C, D and so on. For the universities, we utilized A1, A2, or B1, B2, and other similar designations, and pseudonyms were used for all the selected samples from the institutions.

#### 3.3.1 Population and sampling

The entire academic staff in all the universities were utilized as the population of this research. Since the population is composed of many subgroups that may differ in the characteristics being studied, a probability sampling method known as stratified random sampling is often necessary. This sampling method allows for better coverage of the population and ensures that each subgroup in the population is appropriately represented in the sample (50). To leverage the advantages of the fixed effect model (40), this study stratifies academics based on region and university type.

While earlier published studies, such as that of Lei et al. (36, 40), limited their samples to a developed province in China, this research encompasses a nationwide sample, significantly expanding the scope. This expanded sampling improves the generalizability and relevance of the findings, providing additional support for the earlier results of published studies. The “Seventh Five-Year Plan” which was adopted by the Fourth Session of the Sixth National People’s Congress (51), divided China into three districts—western, eastern and central. The total universities in each district according to the Ministry of Education of China are in 2023 are as follows: Eastern district has 1,164 universities; Central district has 1,034 universities and Western district has 622 universities. However, in each district, based on the countries policy, universities can be categorized into three types: double World-Class universities, vocational colleges, and ordinary universities (52). Therefore, triangulation was ensured by selecting one institution from each type of university in each region, utilizing the simple random sampling method (50). To ensure each university has an equal opportunity to take part, Microsoft Excel was used to select sampled universities randomly (53). As a result, nine universities were selected, one from each type of university, totaling 3 universities from each district.

The complete number of academics in the three eastern universities are 3,371, 1,680 and 643 for double World-Class universities, ordinary universities and vocational colleges, respectively, and the total number of academics for the eastern district amounts to 5,694. Meanwhile, the total number of academics from the central district in each of the three universities—World-Class universities, ordinary universities and vocational colleges, respectively, are 3,512, 1,160 and 658. The total number of academics from the central districts amounts to 5,330. Lastly, the academics from the western district in World-Class universities total 2,683, while the ordinary universities academics are 1,448 and the vocational academics are 521, therefore totaling 4,652 academics from the western district. As such, 16,076 academics serve as the total population of interest for the research. Thus, to determine a sample size to represent each district, we apply the Yamane (54) sampling size formula as follows:

$$n=\frac{N}{1+N{\left(e\right)}^{2}}$$

where *n* represents sample size, *N* denotes the total number of academics, and *e* stands for the significance level or the level of precision. Under normal circumstances, we assume that the precision level is 5%. The results indicated that the sample size for the total number of academics in each district is 373 from eastern district, 372 from central district, and 368 from western district. Subsequently, based on the total number of academics in each sampled university, a proportional allocation is conducted using stratified sampling techniques (42) with simple arithmetic (Sample size in each university = Total academics in each university × Sample size in the district ÷ Total academics in the district). This made it possible to select a specific number of respondents from each university.

Finally, the personnel or HR departments of chosen institutions helped in the random selection of academics for the study, using a method that entailed choosing every fourth person’s name on the list. The aforementioned scholars served both purposes of data gathering and analysis. The samples were chosen at random and then categorized into four groups depending on the academics’ 3-year performance records of panel data: non-performance; low; average; and high. This strategy helped to avoid sampling bias and warranted a random mirror/picture of the job performance groups currently available in universities, as shown in Table 4. Table 5 presents the individual features of the selected sample.

**Table 4:** Sampling and sample observation grouping.

District	University type	Total A.	Sample A.	High P.O.	Average P.O.	Low P.O.	Non-P.O.	Sample A.O.
Eastern	Double World-Class university	3,371	221	117	216	178	152	663
	Ordinary university	1,680	110	65	119	79	67	330
	Vocational college	643	42	24	46	30	26	126
Central	Double World-Class university	3,512	245	136	249	190	160	735
	Ordinary university	1,160	81	51	75	48	69	243
	Vocational college	658	46	29	50	27	32	138
Western	Double World-Class university	2,683	212	93	205	161	177	636
	Ordinary university	1,448	115	58	112	82	93	345
	Vocational college	521	41	23	42	35	23	123
Total		16,076	1,113	596	1,114	830	799	3,339

*N = 1,113; Total A. = Total academics; Sample A., Sampled academics; High P.O., High performance observations; Average P. O., Average performance observations; Low P. O., Low performance observations; Non-P. O., Non-performance observations; Sample A.O., Sampled academics observations.*

**Table 5:** Samples’ demographic profiles.

Control variables	Number	Percentage %
Gender		
Male	532	47.8
Female	581	52.2
Age		
35 or below	284	25.52
36–45	279	25.07
46–55	257	23.09
56 or above	293	26.32
Years of employment		
5 or less	151	13.57
6–10	157	14.11
11–15	171	15.36
11–20	163	14.65
21–25	159	14.28
26–30	159	14.28
31 or above	153	13.75

*N = 1,113.*

### 3.4 Data analysis techniques

Based on the suggestion made by Ahmed et al. (55), SEM is a dominant multivariate technique that can analyze relationships among latent variables and multiple manifest variables, which is helpful in practice, such as in the psychology and management vocations. However, this study processed the relationships between variables by collecting “processed” secondary data (also known as treated data), which does not have latent variables. Specifically, burnout level is an ordered categorical variable, while performance level is a continuous variable, both of which are directly observed without involving complex structures or measurement errors of latent variables (56). Therefore, as suggested by Gordon (57) when dealing with processed data from the secondary treatment panels (especially treated data), multiple regression analysis can help identify key factors that affect the dependent variable and establish predictive models. Furthermore, in order to study the relationships between variables, this study divided respondents into different groups, and group regression will provide the level of significance among the different groups. Convenience for the research, the need for control variables, and the complexity of moderating roles, data are analyzed using a multiple regression model (58).

A multiple regression analysis was undertaken on research question one (RQ1) and the first hypothesis (Ha1), exploring the relationship between job performance and job burnout while taking into consideration temporal and individualistic factors. Group linear regression was used to: (1) ascertain the specific nature of the link between job burnout and their job performance and; (2) prevent mistakes from being introduced based on the heterogeneity of samples. With four job performance observations based on panel data, this made it easier to see how job performance affected job burnout. To bolster the findings of RQ1, performance groups were compared based on the extent of burnout after 3 years.

In order to respond to Ha2, psychological counseling is first evaluated using hierarchical linear regression. This is done to find out if it has a substantial bearing on their job “burnout” and if it acts as a moderator between “job burnout” and their “job performance.” Four types of performance measurements were employed in group regression analysis to validate the first step’s conclusion and prevent sample heterogeneity errors. This was done in an effort to ascertain whether the four types of job performance are moderated by psychological counseling. Moreover, the effect of psychological counseling on “job burnout” and performance group comparisons based on psychological counseling frequency over a three-year period were discussed. The key purpose of this was to examine the RQ2 results in conjunction with the findings of earlier regression analyses. The analysis was done employing version 18 of the Stata software package. Table 6 outlines the research questions, methodology, and assumed hypotheses in detail.

**Table 6:** Research questions and analysis techniques.

Research questions	Hypotheses	Methodology	Analysis
Does job performance influence job burnout among academic staff?	Ha1: When all predictor factors are included, the “job performance” of academics has a significantly negative effect on “job burnout.”	Quantitative	Linear regression, frequency trends
How can a mechanism be developed to address job burnout crises among academics?	Ha2: When all predictor factors are included, academics’ “job performance” and their “job burnout” are significantly moderated by “psychological counseling.”	Quantitative	Linear regression, frequency trends

## 4 Results

The results are presented sequentially based on the stated research questions.

### 4.1 Job performance impacts burnout among academic staff or academics and the impact of their performance on burnout

The outcomes of the panel data (Table 7) in 3-year observations reveal there is an inverse link between academics’ performance and their job burnout (*β* = −0.014, *p* < 0.001), even after adjusting for temporal and individual variables. Thus the results validate Ha1.

**Table 7:** Influence of job performance on job burnout.

Variables	Overall
Gender	−0.061
	(0.043)
Age	0.013
	(0.019)
Years of employment	−0.007
	(0.011)
Job performance	−0.014***
	(0.001)
Constant	2.266***
	(0.125)
Observations	3,339
R-squared	0.043
Individual fixed effect	YES
Time fixed effect	YES

***p < 0.001, **p < 0.01, *p < 0.05.

In order to provide additional evidence for Ha1, the different types of performance were subjected to a group regression analysis, principally to evaluate the extent to which performance level affects job burnout. Table 8 summarizes the results, displaying a substantial and clear inverse association between job burnout and academics’ performance across the four types of performance observations based on the selected 3 years data.

**Table 8:** Influence of job performance on job burnout among four sampled observation groups.

	High P.O.	Average P.O.	Low P.O.	Non-P.O.
Gender	−0.171	0.018	−0.066	0.229
	(0.169)	(0.079)	(0.108)	(0.127)
Age	0.028	0.052	−0.023	−0.073
	(0.065)	(0.036)	(0.057)	(0.058)
Years of employment	0.017	−0.031	−0.036	0.015
	(0.039)	(0.020)	(0.026)	(0.032)
Job performance	−0.225***	−0.114***	−0.226***	−0.101***
	(0.027)	(0.007)	(0.020)	(0.010)
Constant	22.626***	10.395***	16.197***	6.377***
	(2.573)	(0.576)	(1.294)	(0.574)
Observations	596	1,114	830	799
R-squared	0.426	0.490	0.422	0.381
Individual fixed effect	YES	YES	YES	YES
Time fixed effect	YES	YES	YES	YES

***p < 0.001, **p < 0.01, *p < 0.05; High P.O., High performance observations; Average P. O., Average performance observations; Low P. O., Low performance observations; Non-P. O., Non-performance observations.

**Figure 2:**
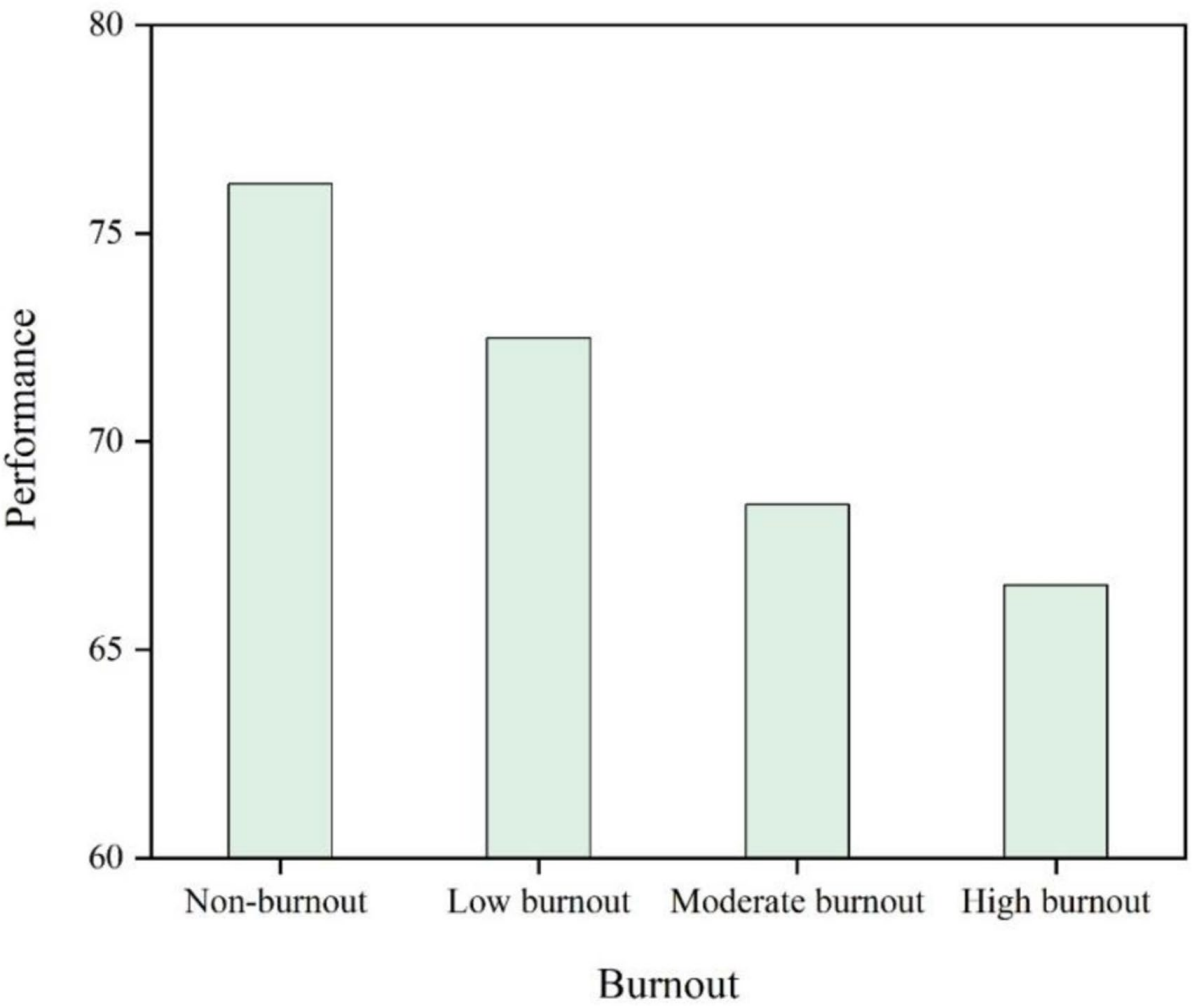
The 3 years comparison of academics’ job performance with dissimilar burnout status.

The study’s aims is two-fold: first, to determine whether job performance and burnout are inversely correlated; second, to reinforce the regression analysis findings that indicate job performance impacts burnout by demonstrating that academic staff with consistently high performance tend to experience low levels of job burnout. In contrast, the non-performance academic observation group—as shown in Figure 2—consistently displays high amount of job burnout.

### 4.2 Job burnout: the function of psychological counseling as a moderator

In the exploration of the moderating impact of psychological counseling on academics who suffer from job burnout, psychological counseling emerges as significantly alleviating the burnout problem. After accounting for the time and individualistic effects, no control factors significantly influence burnout in the initial analysis block. Nevertheless, in the second analysis block, where job performance is included, it has a significant negative effect on predicting burnout (*β* = −0.008, *p* < 0.001). The third analysis block reveals that psychological counseling wields a strong negative effect on job burnout (*β* = −0.172, *p* < 0.001).

In the analysis Block 4, the interaction between psychological counseling and job performance is observed to greatly determine job burnout (*β* = −0.006, *p* < 0.05). As well, Table 9 shows that the relationship between job performance and job burnout is significantly and negatively moderated by psychological counseling.

**Table 9:** Moderating role of psychological counseling on the influence of job performance and job burnout.

	Block 1	Block 2	Block 3	Block 4
Gender	−0.048	−0.053	−0.043	−0.046
	(0.044)	(0.044)	(0.044)	(0.044)
Age	0.024	0.021	0.023	0.020
	(0.020)	(0.020)	(0.020)	(0.020)
Years of employment	0.004	0.003	0.004	0.003
	(0.011)	(0.011)	(0.011)	(0.011)
Job performance		−0.008***		−0.008***
		(0.001)		(0.001)
Psychological counseling			−0.172***	−0.143**
			(0.045)	(0.045)
Job performance *psychological counseling				−0.006*
				(0.003)
Constant	1.659***	2.271***	1.752***	2.304***
	(0.076)	(0.124)	(0.080)	(0.124)
Observations	2,541	2,541	2,541	2,541
R-squared	0.002	0.028	0.013	0.038
Individual fixed effect	YES	YES	YES	YES
Time fixed effect	YES	YES	YES	YES

***p < 0.001, **p < 0.01, *p < 0.05.

Furthermore, the analysis made through the group regression shows that their psychological counseling has a moderating effect on the low (*β* = −0.125, *p* < 0.05) and non-performance (β = −0.054, *p* < 0.05) observation groups after accounting for academics who failed to go to psychological counseling sessions in any of the 3 years (presented in Table 10). Meanwhile in the average performance observation groups (*β* = 0.001, *p* > 0.05) and high-performance observation groups (*β* = −0.004, *p* > 0.05), there is no moderating impact. Hence, there is a noticeable difference between the non-performance, low, average, and high-performance groups. This is particularly the case when discussing the extent to which job burnout is moderated by psychological therapy. The outcomes validated Ha2.

**Table 10:** Moderating role of psychological counseling on the influence of job performance and job burnout among sample observation groups.

	High P.O.	Average P.O.	Low P.O.	Non-P.O.
Gender	0.416	−0.243	−0.314*	0.101
	(0.243)	(0.163)	(0.153)	(0.136)
Age	0.423*	0.031	−0.102	−0.027
	(0.162)	(0.069)	(0.094)	(0.079)
Years of employment	−0.136	−0.015	−0.032	0.074
	(0.100)	(0.036)	(0.035)	(0.046)
Job performance	−0.152*	−0.084***	−0.162***	−0.112***
	(0.057)	(0.016)	(0.026)	(0.013)
Psychological counseling	−0.317	−0.038	−0.563**	−0.194*
	(0.213)	(0.089)	(0.180)	(0.181)
Job performance *psychological counseling	−0.004	0.001	−0.125*	−0.054*
	(0.002)	(0.001)	(0.061)	(0.023)
Constant	17.062*	8.361***	12.561***	7.378***
	(5.527)	(1.238)	(1.655)	(0.808)
Observations	266	477	353	301
R-squared	0.803	0.580	0.740	0.847
Individual fixed effect	YES	YES	YES	YES
Time fixed effect	YES	YES	YES	YES

***p < 0.001, **p < 0.01, *p < 0.05; High P.O., High performance observations; Average P. O., Average performance observations; Low P. O., Low performance observations; Non-P. O., Non-performance observations.

**Figure 3:**
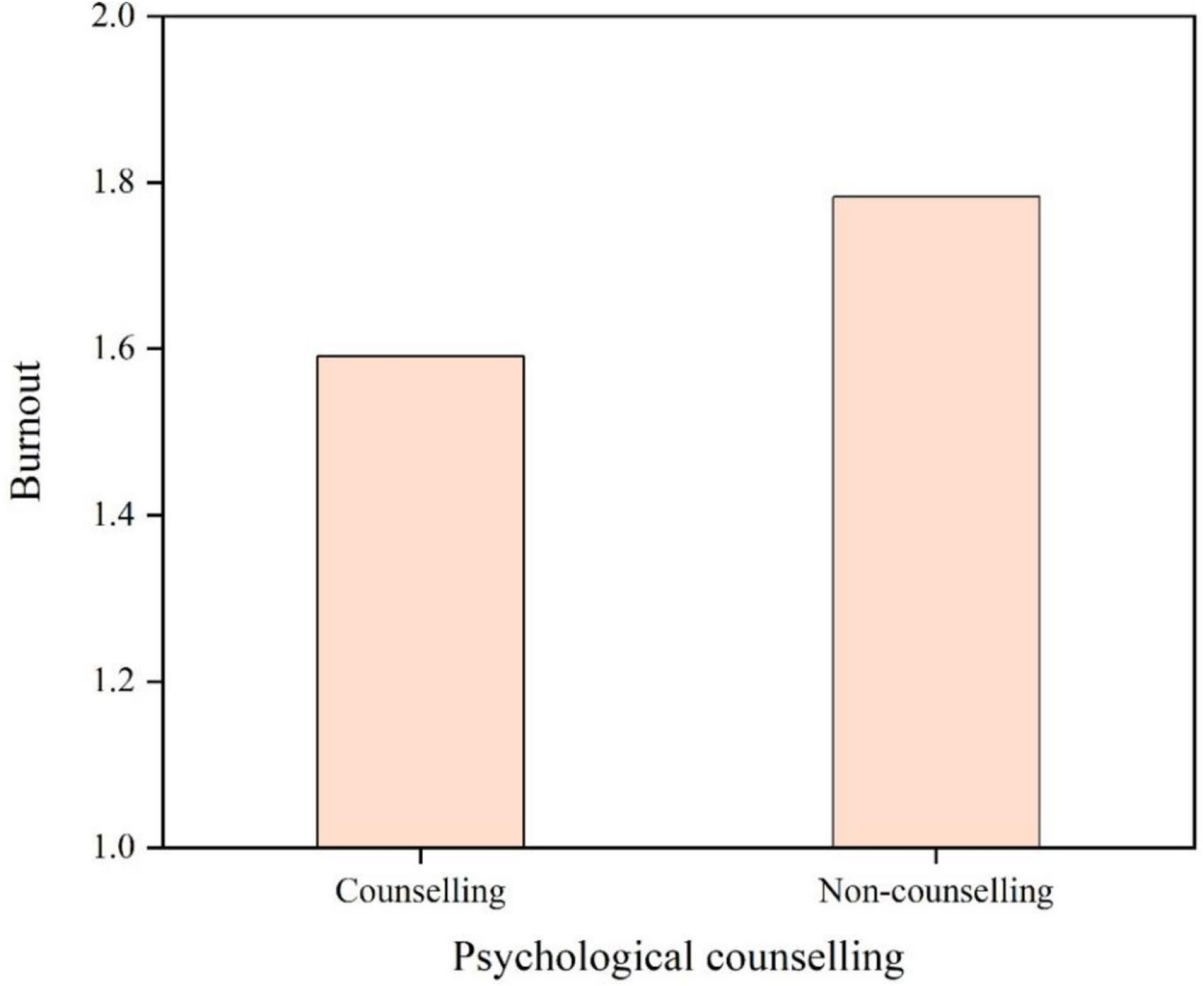
The 3 years (2020–2023) comparison of the role of psychological counseling on job burnout.

The researchers used longitudinal data analysis, as shown in Figure 3, to guarantee accuracy and determine psychological counseling’s moderating influence on their job burnout among the different performance observation groups. The trend is shown from 2020 to 2023. In addition, compared to non-attendees, academics who received psychological counseling displayed reduced levels of burnout.

**Figure 4:**
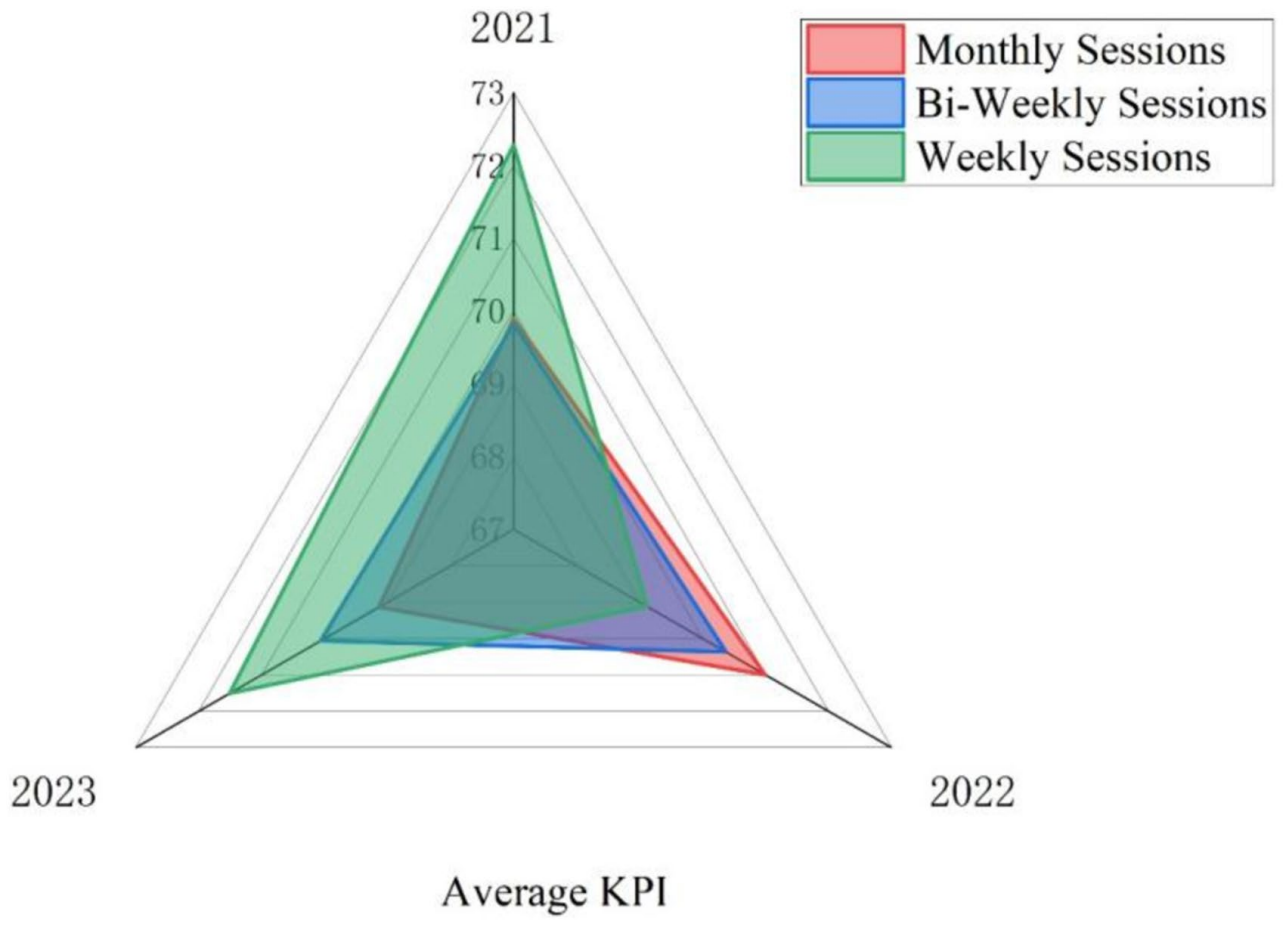
The 3 years (2020–2023) comparison of academics’ KPI performance based on their frequency of psychological counseling.

Likewise, the findings in Figure 4 indicate that academics’ job performance stayed unaffected by the amount or quantity of psychological counseling sessions and interventions performed. This suggests that even though psychological therapy may assist to alleviate academic fatigue, it does not influence job performance.

## 5 Discussion

### 5.1 The association between job-performance and job burnout

According to research by Amer et al. (7), in order to avoid burnout, academics who are highly motivated and productive have a tendency to take proactive steps to address various stressors in their workplace. Contrarily, those who find it difficult to work effectively are more prone to burnout due to their failure to meet job demands (9). Consistent with previous studies by Lei et al. (36, 40), this research found that job performance remains a significant predictor of job burnout, even when controlling for time and individual factors using panel data.

Furthermore, as academics work to improve their performance, they gain greater expertise in their roles and accomplishments. This can result in personal rewards, including promotions within the organization or recognition for their job excellence (44). These findings support the idea that performance can be likened to one of the valuable resources that employees strive to obtain, with the intention to generate better outcomes and ensure less, diminished or preferably no burnout. In this case job performance is the source of the potential reward and a driver of positive outcomes. Employees who strive to do their duties or tasks well are endeavoring to overcome various job stressors in the workplace and therefore avoid burnout. This is consistent with the findings in Bakker and Demerouti (3) who created the Job demands-resources model.

On the other hand, in the Job demands-resources model (3), reducing the impact of job demands and improving the level of job resources can minimize job burnout in the workplace to the greatest extent possible. Research has shown that a decline in job performance can lead to many poor outcomes, such as emotional exhaustion (59), and diminished personal achievement (60), both of which are important dimensions of job burnout (3). Therefore, after becoming an academic, job performance plays a significant role in improving job resources and reducing job demands.

### 5.2 Impact of psychological counseling as a procedure for treating their burnout: utilizing post-cautionary or pre-cautionary measurement

Psychological counseling as a post-measurement has been shown to be useful in minimizing job burnout, as revealed by the results of this study. This finding generally agrees with previous literature on the efficacy of psychological counseling in mitigating job burnout across various contexts (39, 40). In addition, the results provide concrete proof that psychological counseling wields a mitigating impact on both non-performing and low performance groups.

The association between job performance and their job burnout of the average and high-performance observation groups is not mediated by psychological counseling. This is predicated on the discovery pertaining to RQ1, which implies that academics who underperform are more susceptible to burnout and more inclined to seek support through counseling services, as a consequence. In contrast, people who perform well typically experience far less burnout. Consequently, there is no longer a need for psychological counseling assistance. Consequently, the importance of psychological counseling as a subsequent approach to addressing their job burnout concerns continues to be significant.

Moreover, the correlation between job performance, psychological counseling, and job burnout as shown by this research study suggests that persons who are suffering from burnout may suffer from recurring attacks. Even if they seek to alleviate burnout through psychological counseling. It is important to acknowledge that while psychological counseling may alleviate burnout, as indicated by previous studies (39, 40) and in these results, it cannot enhance performance. Consequently, academics might become caught in a repetitive pattern where poor performance leads to frequent burnout and situations wherein the outcome of job demands prove to be pervasive, such as stress and emotional exhaustion (3). As academic personnel, relying solely on post-measurement for their job burnout therapy in one’s career may not be sufficient to achieve a permanent cure and may in fact lead to various onerous job demands. For this reason it is crucial to examine and highlight the significance of pre-measurements.

## 6 Theoretical and practical implications

The findings contribute to the existing literature on the research of job burnout by exploring how the relationship between academics’ job performance and their burnout affects different levels of teaching staff, offering practical insights. Firstly, this research contributes significantly to the area of job burnout studies by expanding upon existing knowledge and presenting a fresh perspective on the topic. The theoretical contributions made by this study are both legitimate and substantial. Secondly, while most research typically focuses on reducing employee burnout to improve academics’ performance (61, 62), this study introduces a novel methodological approach to explore the relationship between these two variables.

In a period marked by the rapid expansion of higher education, there is a common misconception, particularly in developing nations, that being employed as academics entails a much easier workload and minimal dedication to their profession (63). This assumption and attitude should be completely dismantled before and subsequent to when an employment contract begins. Based on this, the notion that “prevention is better than cure” should be acted on, as suggested by Kennedy (64). However, in most universities the academic recruitment processes which are so important for their viability have either ignored, underestimated or paid little attention to this adage, and this helps to explain why there is much scholarly debate or discussion on higher education and examples of job burnout (65). Consequently, in order to reduce academics’ job burnout after receiving a contract, it is recommended to create proactive recruitment strategies for academics as a preventive strategy. It should function in such a way to mitigate their job demands in the workplace, increase job resources, and detect any signs of job burnout crisis that can then be dealt with. Essentially, universities should prioritize hiring academics who exhibit good to excellent competency, resilience to stressful situations and high job performance in a fair performance evaluation system so that symptoms of burnout are prevented.

On the other hand, since counseling is not primarily intended to enhance job performance, it does not inherently result in skilled, revitalized, motivated, enthusiastic, and proficient staff. Based on these discoveries, it is recommended to evaluate the cognitive, educational, and psychological aspects of potential employees in addition to comprehensively assessing the abilities of all applicants at the human resources recruitment process. Put differently, if universities desire to hire capable, self-driven, mentally strong and enthusiastic individuals who are prepared to work as academics, it is imperative for their recruitment procedures to include psychological counseling examinations so that the applicant’s competency skills can be thoroughly assessed.

Applicants must undergo a thorough evaluation to determine their capacity for intense and diligent labor. Additionally, their psychological fit for the academic role should be analyzed by examining their motivations, passions, and determination. After recruiting the right individuals, strategies for ongoing professional development that include both academic and psychological components must be established. Research has shown that learning opportunities—as one of the important job resources—can buffer job demands, such as one of the dimensions of job burnout-emotional exhaustion and reduce job burnout (66). This will equip them with the essential skills to effectively adapt to the ongoing transformations within the higher education system, especially in response to the swift technological advancements currently taking place. Additionally, the university administration division ought to enhance its training initiatives with the goal of enhancing staff members’ performance and competence. This involves identifying skills gaps and promoting mental wellbeing among staff members.

## 7 Limitations and future studies

While this analysis deployed a novel approach to assess the impact of job burnout on staff members’ performance, it did have some limitations. Initially, this study suggested that psychological assessments should be incorporated into recruiting processes, however, it did not specify the specific psychological criteria to be employed in these assessments. Furthermore, although panel data is sampled and used throughout China, the applicability of the results may be limited solely to that country, potentially limiting the applicability of its findings to other nations. Furthermore, studies can explore mediating and moderating variables in the hypothesized association between performance and burnout. This will further enhance the robustness of the measurement method/path, and it could lead to interesting findings.

To attain a more inclusive and generalizable understanding of the influence of job burnout, further research could broaden the inquiry to diverse contexts and a replica of this study can also be conducted in private universities due to the dynamics of their organizational management. It should be noted that this study utilized secondary data for its analysis. This study used processed (treated) secondary data from the treatment panel at the secondary sources; it therefore furthered processing them with AMOS for the usage of SEM, which was rather restricted. However, this research made a gateway developing pathways and variables for future research. Hence, future research can collect primary data to test the correlation between job performance and burnout by using the Structural Equation Modeling. Likewise, other statistical methods and packages such as AMOS, SPSS can be used to investigate the data.

## 8 Conclusion

The recent research done in China by Lei et al. (35, 39) is not inherently refuted by this study. What it does do is supplement and complement a wider nationwide dataset by adopting a distinct form of analysis. Moreover, the results and analysis discussed in this paper have considered a multifaceted view to draw a conclusion that is comparable to the studies conducted recently. Nevertheless, the research results indicate that in academic environment, academics’ burnout is influenced by their performance. It is obvious that academics’ competence deserves more attention, as the operations of universities rely heavily on academics’ expertise and ability to do their job, such as teaching, administrative tasks dealing with their subjects, and research. By ensuring personal abilities and enhancing individual performance to reduce job burnout, pre-measurement can be used instead of post-measurement, because the former will benefit the stability of academic staff. In addition, since psychological counseling moderates the relationship between job performance and job burnout, the research findings confirm that psychological counseling effectively addresses job burnout. However, what is also expressed is that doubts exist about its ability to improve the performance of underperforming groups and/or transforming them into high-performing groups. On the one hand, universities need to provide professional psychological counseling services to academic personnel for continuous professional development and personal wellbeing. On the other hand, in order to reduce job burnout, institutions should strengthen their recruitment strategies by employing psychologists who will assess candidates’ cognitive, social, and emotional intelligence and stability. This method can be more effective than relying solely on education, skills, and medical evaluation.

## Data Availability

The data analyzed in this study is subject to the following licenses/restrictions: further information and data set can be provided with further request. Requests to access these datasets should be directed to gs61670@student.upm.edu.my.
